# Supplementary material for: Evaluation of approaches to strengthen civil registration and vital statistics systems: A systematic review and synthesis of policies in 25 countries
Source: PLoS Med. 2019 Sep 27;16(9):e1002929. doi: 10.1371/journal.pmed.1002929 (PMC6764661; doi:10.1371/journal.pmed.1002929)
Supplement: S1 Text — (PDF) [file pmed.1002929.s001.pdf]

Towards universal civil registration and vital statistics systems: a systematic review and meta-analysis of policies to improve birth and death registration

DRAFT

Introduction

Civil Registration and Vital Statistics (CRVS) systems register vital events including births, deaths, and causes of deaths in countries [1]. Civil registration and supporting legal documentation helps establish citizenship, allows citizens to exercise a broad range of rights, and facilitates access to essential services including social welfare, education, health, and legal protection. Systematic collection of registration data also improves demographic information necessary for governance. For example, birth and death data can be used to inform population growth and movement and inform fiscal policy. Within health, functioning CRVS systems can estimate national and subnational burden of disease, the impact of different disease programmes, the cost-effectiveness of disease interventions, and health service needs and coverage [2]. To date, many countries have underdeveloped CRVS systems and have had to rely on ad-hoc studies, surveys, and modelling for these essential information [3-5]. Unfortunately, many of these data sources are time-limited and externally supported. There is a need for indigenous and sustainable CRVS systems to generate strategic information to improve public health planning, budgeting and programming.

Civil registration is defined as the continuous, permanent, compulsory and universal recording of the occurrence and characteristics of vital events in accordance with the legal requirements in each nation. Vital events captured in CRVS systems include the registration of births, deaths (including cause of death), marriages, adoptions, and divorces. Public health authorities primarily focus on registration of births, deaths, and causes of deaths for decision making. Global guidelines have been useful in establishing CRVS norms and standards for countries. The Statistical Commission of the United Nations provides comprehensive guidance on how CRVS systems can achieve universal coverage, continuity, confidentiality, and regular dissemination in order to be a dependable and primary source of vital statistics [6]. Other technical guidance covers CRVS system strategic planning, legal frameworks, registration practices, death certification and cause of death, International Classification of Diseases (ICD) data quality, interim methods for vital statistics, and how to build political and community support for CRVS systems [7].

All countries have agreed to achieve the Sustainable Development Goals that specify targets related to CRVS, including (1) by 2030, provide legal identify for all, including birth registration and (2) by 2020, enhance capacity-building support to developing countries to increase significantly the availability of high-quality, timely and reliable disaggregated data [8]. Although there has been progress in CRVS system development over the past two decades, birth and death registration rates continue to increase at a slow rate [9-11]. Worldwide, the proportion of deaths registered increased from 36% in 2000 to 38% in 2015

DRAFT

whilst the proportion of children under five with a registered birth increased from 58% to 65% [12]. Moreover, independent assessments indicate that the majority of registered deaths have issues surrounding the quality of cause-of-death ascertainment [13]. For example, the latest data available from WHO indicate that while an average of 86% of deaths included cause of death, 30% of cause-of-death reports used ill-defined ICD codes [14]. Increasing death registration rates is critical because cause-of-death data would become more representative and could potentially be used at the subnational level. Although significant progress has been made in evaluating the role of information technology interventions in CRVS systems, other interventions lack formal reviews and evaluations [15,16]. We aim to systematically review and meta-analyse the evidence on policy interventions to improve birth and death registration.

Methods

Study conduct

This systematic review will be conducted in accordance with the Preferred Reporting Items for Systematic Review and Meta-Analysis (PRISMA) guidelines [17]. The ABI/INFORM, Embase, JSTOR, PubMed, and WHO Index Medicus databases will be systematically searched without language, publication, or any other limits. Given that the Statistical Commission of the United Nations adopted the *International Programme for Accelerating the Improvement of Vital Statistics and Civil Registration Systems* to assist countries with incomplete registration or entirely lacking a CRVS system in 1991, we included articles implemented and published from 1991 onward [18]. All sources cited in the 2007 and 2015 CRVS Lancet series will be reviewed for inclusion [1,12,19-24]. CDC's International Institute for Vital Registration and Statistics, United Nations Children's Fund (UNICEF), UN Statistics, World Bank, and World Health Organization (WHO) will be contacted for relevant publications on CRVS systems. Other stakeholders working on CRVS system strengthening will also be contacted to identify evaluations of system improvement interventions.

Search strategy and selection criteria

The search strategies will be designed with a librarian to identify studies that include CRVS system evaluations. Per recommendations from the PRISMA Group, eligibility criteria will be based on key study characteristics: population, intervention, comparator, outcome, and design [17]. Specifically, sources will be included when (1) they include a population eligible for birth and/or death registration, (2) the intervention is a new policy (i.e. novel legislation or change in programme implementation designed to improve birth and/or death registration), (3) the comparator includes the lack of the new policy, (4) the outcome is birth registration rate, death registration rate, timeliness of birth registration, timeliness of

DRAFT

death registration, and/or operational considerations (i.e. acceptability to persons registering births or deaths, acceptability to staff managing and implementing birth or death registration, human resource requirements, costs to the health system, adverse events, and/or facilitators or barriers learnt during implementation), and (5) the study design is cross-sectional, cohort, case-control, or randomised controlled trial. Studies describing operational considerations will not require a comparator arm to be included.

Study screening and extraction

Two investigators will independently screen titles of all identified articles. Abstracts from relevant titles will then be screened. The investigators will then match the full texts of all articles selected during abstract screening against the inclusion criteria. Disagreements will be resolved through discussion with a third investigator. References for included studies will be reviewed for additional reports. Articles failing to meet these inclusion criteria will be excluded from this review. Two investigators will complete the data extraction using a standardised extraction form comprising four tables. The first table will summarise the study setting, the second table will summarise study design, the third table will summarise quantitative outcomes, and the fourth table will summarise operational considerations.

Quality assessment

The Newcastle-Ottawa Quality Assessment Scale will be used to assess bias in studies [25]. This scale rates studies based on eight criteria in evaluating selection bias, confounding, and measurement bias.

Statistical analyses

Differences in registration rates will be entered as proportions and stabilised using the Freeman-Tukey-type arcsine square-root transformation [26]. If interventions are similar enough to combine, the differences in registration rates with and without the intervention will be pooled based on based on policy mechanism (e.g. supply, demand, requirement for legal services, punishable by law, incentives, and combination) [27]. Fixed-effect models assume that the magnitude and direction of an interventions' effects are identical across the sources and that observed differences among results are due solely to chance [28]. Random-effects models assume that the magnitude and direction of an interventions' effects are not identical but follow a distribution [28]. Since it is possible that the registration rate differences could differ for reasons other than chance, random-effects models will be used for all analyses. An I-squared statistic will be used to measure heterogeneity [29]. If there is moderate to significant heterogeneity in estimates, potential causes will be explored. STATA will be used for all quantitative analyses.

DRAFT

DRAFT

References

1. AbouZahr C, de Savigny D, Mikkelsen L, Setel PW, Lozano R, Lopez AD. Towards universal civil registration and vital statistics systems: the time is now. *Lancet* 2015; **386**(10001): 1407-18.
2. Mortality GBD, Causes of Death C. Global, regional, and national life expectancy, all-cause mortality, and cause-specific mortality for 249 causes of death, 1980-2015: a systematic analysis for the Global Burden of Disease Study 2015. *Lancet* 2016; **388**(10053): 1459-544.
3. Ye Y, Wamukoya M, Ezeh A, Emina JB, Sankoh O. Health and demographic surveillance systems: a step towards full civil registration and vital statistics system in sub-Sahara Africa? *BMC Public Health* 2012; **12**: 741.
4. Sankoh O, Byass P. The INDEPTH Network: filling vital gaps in global epidemiology. *Int J Epidemiol* 2012; **41**(3): 579-88.
5. Principles and Recommendations for a Vital Statistics System. New York: United Nations, 2014.
6. Strengthening civil registration and vital statistics for births, deaths and causes of death: resource kit. Geneva: World Health Organization, 2012.
7. United Nations General Assembly Resolution 70/1. Transforming our world: the 2030 Agenda for Sustainable Development. 2015. http://www.un.org/ga/search/view_doc.asp?symbol=A/RES/70/1&Lang=E (accessed 4 November 2015.
8. Abouzahr C, Azimi SY, Bersales LGS, et al. Strengthening civil registration and vital statistics in the Asia-Pacific region: Learning from country experiences. *Asia-Pacific Population Journal* 2014; **29**(1): 39-73.
9. Every Child's Birth Right: Inequities and trends in birth registration. New York: United Nations Childrens Fund, 2013.
10. Muzzi M. UNICEF Good practices in integrating birth registraiton into health systems (2000-2009), Case Studies: Bandladesh, Brazil, the Gambia and Delhi, India. New York: United Nations Children's Fund (UNICEF), 2009.
11. Mikkelsen L, Phillips DE, AbouZahr C, et al. A global assessment of civil registration and vital statistics systems: monitoring data quality and progress. *Lancet* 2015; **386**(10001): 1395-406.
12. Phillips DE, Lozano R, Naghavi M, et al. A composite metric for assessing data on mortality and causes of death: the vital statistics performance index. *Population Health Metrics* 2014; **12**(14).
13. Observatory GH. Civil registration coverage. 2013 (accessed 2/12/2016.
14. Move it: Report on Monitoring of Vital Events using Information Technology. Geneva: World Health Organization, 2013.
15. Systematic review of eCRVS and mCRVS interventions in low and middle income countries. Geneva: World Health Organization, 2013.
16. Liberati A, Altman DG, Tetzlaff J, et al. The PRISMA statement for reporting systematic reviews and meta-analyses of studies that evaluate health care interventions: explanation and elaboration. *J Clin Epidemiol* 2009; **62**(10): e1-34.
17. United Nations. International Programme for Accelerating the Improvement of Vital Statistics and Civil Registration Systems. In: Office UNS, editor. New York, New York, USA; 1989.
18. Phillips DE, AbouZahr C, Lopez AD, et al. Are well functioning civil registration and vital statistics systems associated with better health outcomes? *Lancet* 2015; **386**(10001): 1386-94.
19. AbouZahr C, de Savigny D, Mikkelsen L, et al. Civil registration and vital statistics: progress in the data revolution for counting and accountability. *Lancet* 2015; **386**(10001): 1373-85.
20. Mahapatra P, Shibuya K, Lopez AD, et al. Civil registration systems and vital statistics: successes and missed opportunities. *Lancet* 2007; **370**(9599): 1653-63.
21. Hill K, Lopez AD, Shibuya K, Jha P, Monitoring of Vital E. Interim measures for meeting needs for health sector data: births, deaths, and causes of death. *Lancet* 2007; **370**(9600): 1726-35.

DRAFT

22. Setel PW, Macfarlane SB, Szreter S, et al. A scandal of invisibility: making everyone count by counting everyone. *Lancet* 2007; **370**(9598): 1569-77.
23. AbouZahr C, Cleland J, Coullare F, et al. The way forward. *Lancet* 2007; **370**(9601): 1791-9.
24. Wells G, Shea B, O'Connell D, et al. The Newcastle-Ottawa Scale (NOS) for assessing the quality of nonrandomised studies in meta-analyses: The Ottawa Hospital, 2011.
25. Freeman MF, Tukey JW. Transformations related to the angular and the square root. *The Annals of Mathematical Statistics* 1950; **21**(4): 607-11.
26. Cooper H, Hedges L. The Handbook of Research Synthesis. New York, NY: Russel Sage Foundation; 1994.
27. Collaboration TC. Cochrane Handbook for Systematic Reviews of Interventions; 2011.
28. Higgins JP, Thompson SG. Quantifying heterogeneity in a meta-analysis. *Stat Med* 2002; **21**(11): 1539-58.

DRAFT
