# Supplementary material for: Evaluation of approaches to strengthen civil registration and vital statistics systems: A systematic review and synthesis of policies in 25 countries
Source: PLoS Med. 2019 Sep 27;16(9):e1002929. doi: 10.1371/journal.pmed.1002929 (PMC6764661; doi:10.1371/journal.pmed.1002929)
Supplement: S3 Text — (PDF) [file pmed.1002929.s003.pdf]

Search strategy developed by CDC Librarian. Search conducted 27 January 2017.

(1) (civil OR birth* OR death* OR mortality OR vital*) ADJ2 (registr* OR certificat* OR notif* OR record* OR report* OR collect* OR statistic*)

(2) legislation OR policy OR law OR laws OR legal* OR system OR systems OR administrat* OR innovat* OR incentive OR penalty OR program OR programme OR programs OR programmes OR intervention* OR campaign* OR funeral OR health OR burial OR mortuar* OR mortician OR inheritance OR insurance OR school OR enrollment OR welfare OR social OR immuni* OR vaccin* OR citizen* OR govern* OR valid* OR complete* OR coverage* OR lesson* OR develop* OR identity* OR responsib* OR right* OR strengthen* OR barrier*

(3) Search number (1) and (2)

(4) (laboratories or patients or neoplasms or tuberculosis or cells or biology or therapeutics or viruses or infection or "wounds and injuries" or "surgical procedures, operative" or "general surgery" or "influenza, human" or mosquito* or volvulus or homicid* or embolism* or trisomy or fish or cattle or particulate matter or air pollution or bereavement or traffic accident* or neuromuscular blocker* or aneurysm* or embolism* or anaphylaxis or tumor* or tumour* or schizophreni* or gastrostomy or obesity or drown* or tsunami* or occupational therapy or drug-related or medication* or anesthesia or overdos* or stroke or strokes or cardiac or myocardial or infarction* or vasculidities or transplantation* or arthritis or ulcer* or colitis or preanesthesia or opioid* or fracture* or antidepressant* or cystic fibrosis or aortic or ventricle or acidosis or hip or knee or arthroplasty)

(5) Search number (3) NOT (4)
