# Supplementary material for: Evaluation of approaches to strengthen civil registration and vital statistics systems: A systematic review and synthesis of policies in 25 countries
Source: PLoS Med. 2019 Sep 27;16(9):e1002929. doi: 10.1371/journal.pmed.1002929 (PMC6764661; doi:10.1371/journal.pmed.1002929)
Supplement: S1 Table — (PDF) [file pmed.1002929.s004.pdf]

Individual study quantitative results

Article	Country	Policy	Geographic scale	Years of implementation (I), comparator result (CR), and intervention result (IR)	Vital events measured	Comparator used	Comparator result	Intervention result
Garenne, 2016	South Africa	Supply, Demand, Incentive	Agincourt (rural area)	I: 1992-2014, CR: 1992, IR: 2014	Births and deaths	Baseline year	7.8% births; 51.4% deaths	90.5% births; 97.1% deaths
Dababneh, 2015	Jordan	Supply	National	I: 2003 on, CR: 2004, IR: 2011	Deaths	Death notification forms received by MOH as a percentage of forms reported to the Civil Status and Passports Department	71.9%	77.7%
Joubert, 2013	South Africa	Supply	National	I: 1992 on, CR: 1998, IR: 2007	Deaths	Baseline year	73% male deaths; 63% female	87% male deaths; 79% female
Kabadi, 2013	Tanzania	Supply	Rufiji district	I: Sep 2012-Mar 2013, CR: Sep 2011-Feb 2012, IR: Sept 2012-Feb 2013	Births and deaths	Baseline period	564 births notified (28% of all births), 285 births registered (51% of notified births); 29 deaths notified (2% of all deaths), 13 deaths registered (45% of notified deaths)	1,048 births notified (51% of all births), 312 births registered (30% of notified births); 165 deaths notified (14% of all deaths), 19 deaths registered (12% of notified deaths)
Ozdemir, 2015	Turkey	Supply	National, Izmir	I: 2001-2013, CR: 2001-2008, IR: 2009-2013	Deaths	Baseline period	National: 57.7% males 61.1% females; Izmir: 80.0% males 72.4% females	National: 99.2% males 97.0% females; Izmir: 103.9% males 102.0% females
Robertson, 2013	Zimbabwe	Incentive	Manicaland	I: 2010-2011	Births	Control group	45% (163/366)	48% (255/528) UCT; 60% (252/417) CCT
Fagernas, 2013	Ghana	Supply, Demand	National	I: 2004-2005, CR: 2003, IR: 2008	Births	Baseline year	44%	71%

Singh, 2012	India	Supply	Haryana	I: 2005-2009, CR: 2004, IR: 2009	Births and deaths	Baseline year	70% births; 73.5% deaths	95% births; 92.1% deaths
Singogo, 2013	Malawi	Supply	Traditional Authority Mwambo, Zomba district	I: 2007-2011, CR: 2011, IR: 2011	Births and deaths	Maternity registers (for birth events only)	6397 births	1753 births
Upham, 2012	South Africa	Supply, Demand, Incentive	National	I: 1997-2004, CR: 1997, IR: 2004	Births and deaths	Baseline year	63% deaths	82% deaths; almost universal births
Mony, 2011	India	Supply, Demand	Five subdistricts in southern India	I: Sep 2007-Aug 2008, CR: 2007, IR: 2008	Births and deaths	Baseline year	50% births; 25% deaths	80% births; 61% deaths
UNICEF, 2010	Bangladesh	Supply, Demand, Incentive	National	I: 1996 on, CR: 1996, IR: 2006 and 2008	Births	Baseline year	7%	10% in 2006; 40% in 2008
UNICEF, 2010	Brazil	Supply, Demand, Incentive	National	I: 1997-2008, CR: 1995, IR: 2007	Births	Baseline year	69.7%	87.8%
UNICEF, 2010	Gambia	Supply, Demand	National	I: 1996-2006, CR: 2000, IR: 2006	Births	Baseline year	32%	55%
Rao, 2004	South Africa	Supply, Demand	National	I: 1990 on, CR: 1990, IR: 1999-2000	Deaths	Baseline year	54%	89%
Woodward, 2003	Canada	Penalty	Ontario	I: 1996, CR: 1991, IR: 1996	Births	Baseline year	99.4%	99%
UNICEF, 2013	Brasil	Supply, Incentive	National	I: 1999-2008 CR: 1998 IR: 2008	Births	Baseline year	64%	91%
