# Supplementary material for: Evaluation of approaches to strengthen civil registration and vital statistics systems: A systematic review and synthesis of policies in 25 countries
Source: PLoS Med. 2019 Sep 27;16(9):e1002929. doi: 10.1371/journal.pmed.1002929 (PMC6764661; doi:10.1371/journal.pmed.1002929)
Supplement: S2 Table — (PDF) [file pmed.1002929.s005.pdf]

Individual study operational considerations

Article	Policy	Intervention timeframe	Type of operational consideration	Description
Curioso, 2013	Supply	2012-2013	2) Human resource requirements 3) IT or infrastructure requirements 6) Facilitators 7) Barriers	The major challenge for real-time registration was internet connectivity at the health facilities. Appropriate training of human resources, especially in office automation, health information systems, and change management was critical to the success of this policy.
Duff, 2016	Supply	1992-2014	5) Adverse events 7) Barriers	Barriers to successful national policy success were varied implementation by region and hidden fees that some parents still had to pay in spite of the new legislation.
Fagernas, 2013	Supply, Demand	2013	4) Costs to the health system	The authors state that the component of integrating birth registration activities into health care services in the community reduced indirect costs of registration.
Gadabu, 2014	Supply	2003 on	2) Human resource requirements 3) IT or infrastructure requirements 7) Barriers	Given the literacy level of the village headman was low, the project relied on literate members of the community to assist the headman and use the touchscreen computer. Maintaining a connection for data flow was challenging. Relying on past data on paper-based vital event registration to gauge coverage was not possible due to low registration rates with paper-based systems.
Garenne, 2016	Supply, Demand, Incentive	Mar 2013	2) Human resource requirements 3) IT or infrastructure requirements 6) Facilitators	The parallel development of IT, infrastructure, and human resources facilitated policy success. A major facilitator to the interventions to improve CRVS is that the government at the same time started requiring birth certificates for obtaining a National ID. Another facilitator was the new political will for registration for all social/racial strata after the 1994 elections
Hunter, 2011	Incentive	1992 on	7) Barriers	Many poor families were not able to acquire a birth certificate for Bolsa Familia due to limited access, education, or awareness. In order to confront this barrier, the Ministry of Justice began registration campaigns in underserved areas of the country.
IDB, 2009	Supply, Demand	Sep 2012-Mar 2013	2) Human resource requirements 3) IT or infrastructure requirements 6) Facilitators	Peru's CRVS coordination is successful in part to its simultaneous development of infrastructure and training of staff at all levels. Another reason for the program's success is that it is autonomous of the Peruvian government. The authors argue that the coordinating entity is not required to answer to political demands, it has retained its focus on technical aspects of civil registration and has been able to constantly improve its capacity to deliver services.
Kabengele, 2014	Supply	2005-2010	2) Human resource requirements 3) IT or infrastructure requirements 6) Facilitators	Overall this expansion of registration services was low cost and required minimal human or computer resources
Lhamsuren, 2012	Demand	1994	1) Acceptability to staff	The nurses reported high satisfaction and motiviation with their job because of their ability to help families with multiple problems and interact face to face. However, one negative consideration is that nurses desired a larger incentive to conduct their household visits since the work was time consuming and strenuous in addition to their normal responsibilities.
Lu, 2002	Supply	2004-2006	4) Costs to the health system 6) Facilitators	Although they filled a void in a setting where most deaths occurred at home, the price of full-time home-based death certifiers was higher than the certification at hospitals. This was because hospital staff with other responsibilities could also certify events.

Modi, 2016	Supply	2000	1) Acceptability to staff 2) Facilitators to intervention implementation	The staff found the application to be "acceptable, feasible, and useful." Having a relatively high proportion of facility-based births (87%) made the impact of this mobile platform used by hospital staff large.
Mony, 2011	Supply, Demand	1997-2004	1) Acceptability to staff 6) Facilitators 7) Barriers	Utilising multiple interventions accounted for the policy's effectiveness. The policy spread across community, government, and household levels. Creating partnerships between government and local non-governmental organizations facilitated interaction with the public to encourage and educate about civil registration. Finally, integrating diverse professions into the civil registration process caused initial confusion about the distribution of roles and responsibilities amongst professional staff. The lack of a computerised system and technology-based training made the intervention less effective and efficient.
Ohemeng-Dapaah, 2010	Supply, Demand	2013 on	2) Human resource requirements 3) IT or infrastructure requirements 6) Facilitators	The two most crucial factors to the programs success were adequate infrastructure and health workforce. During implementation of this new policy, Ghana expanded access to the health sector. Registration rates increased further as more births and deaths occured in health facilities.
Prata, 2012	Supply, demand	2001-2013	2) Human resource requirements 4) Costs to the health system	The authors note that this community health worker strategy is more sustainable than requiring additional staff because it leverages already exisiting human resources and health systems. However, the fact that these workers are currently unpaid may pose a greater problem when scaling up the intervention.
Prybylski, 1992	Supply	2001 on	6) Facilitators	Mothers were incentivised to register their birth because of reduced cost of antenatal and immunisation services associated with a birth ceritificate.
Silva, 2016	Supply	1988	2) Human resource requirements 4) Costs to the health system	The average annual running cost of the CHW intervention per 1,000 population was $523 ($72 per vital event reported). One major challenge was the size of the geographic area to which each CHW was assigned. These CHWs were overburdened and sometimes required to respond to other duties in the area.
Silva, 2016	Supply	Jan 2012-Mar 2013	4) Costs to the health system	The average annual running cost of the CHW intervention per 1,000 population was $6,344 ($149 per vital event reported). The investments and resources in Mali proved a challenge. It was financially difficult to provide incentives and routine supervision/monitoring at all levels.
Singh, 2012	Supply	Jan 2010-Dec 2013	1) Acceptability to staff	Medical staff at PHCs reported that their registration responsibilities were difficult because they were not trained or educated in legal services. Nurses and health workers considered the work burdensome. For these reasons, initial adoption of the new system was challenging. The authors state that within the first year of implementation, frequent training increased the acceptability to staff.
Singogo, 2013	Supply	2010-2011	2) Human resource requirements	The two major challenges with implementing paper-based registration was insuffificent supervision of the village headpersons to ensure complete and high quality documentation of community vital events, and illiteracy among village headpersons
Skiri, 2012	Supply	2004-2005	2) Human resource requirements 3) IT or infrastructure requirements	Two major challenges to implementation were unstable power supplies and staff with limited computer technology skills.
Starr, 1995	Supply	Jul 2012-Sep 2013	1) Acceptability to staff 3) IT or infrastructure requirements 4) Costs to the health system	Officials from 31/50 states said that the digitisation improved timeliness of data flow from hospitals to the state office. Officials from 33/50 states said accuracy of data was improved. Cost lowered in some states but increased or remained the same in others. Personnel training and technical complications posed problems during implementation. Despite having electronic systems, many states still required paper forms. Timeliness of reporting at the federal level did not improve.

Tangcharoensathien, 2014	Incentive	2005-2009	6) Facilitators	The unique citizen identification number was introduced in 1982 and assigned to all citizens at birth. This helped facilitate registration and transitioning to a computerized system.
Toivanen, 2011	Supply	2009-2010	3) IT or infrastructure requirements	The largest barrier was the limited capacity for technology use. For example, Liberia lacked government ownership of the new technology because of capacity issues. This highlights the need for ICT capacity building in order to implement sustainable systems.
Tripp, 2015	Supply	2010-2011	1) Acceptability to staff 2) Facilitators to intervention implementation	Uptake of the electronic system was greater in funeral directors and registrars than doctors, because they have a personal/financial investment in submitting timely death certificates. The electronic system was not well accepted by physicians, who found the paper-based sytem more efficient.
UNICEF, 2010	Supply, Demand, Incentive	2007-2011	6) Facilitators	The authors note that the effort to improve birth registration was successful because of the use of multiple interventions.
UNICEF, 2010	Supply, Demand, Incentive	1997-2004	4) Costs to the health system 6) Facilitators 7) Barriers	The initial mandate to provide monetary incentives to registrars did not work because heterogeneity in implementation at the state level. Some states never adopted the mandate at all. Monetary incentives were much more successful when provided directly by the Ministry of Health. Additionally, integration of civil registration into the maternity wards proved costly in areas where few children are born each day. In these cases, maintaining the civil registration unit was costly and facilities did not receive incentives very often to cover these costs. Moreover, during integration maternity ward staff were often unaware of the civil registration law, new monetary incentives, and their mandate to perform civil registration. In summary, the monetary incentives were only successful when implemented within a more comprehensive framework for registration improvement, more collaboration and training for the health sector, and involvement at all levels implementation.
UNICEF, 2010	Supply, Demand	2002-2004	7) Barriers	Although new legislation made birth registration free, many facilities still charged for birth certificates. Additional barriers were cost of transport and taking time off work to visit the office.
UNICEF, 2010	Supply	Sep 2007-Aug 2008	6) Facilitators 7) Barriers	One of the crucial factors for Delhi's success is leveraging existing systems such as immunisation to improve civil registration with little additional cost to the health care system. However, one of the major challenges is that the system was strictly built to only serve permanent Delhi residents because of the legal requirement that vital events can only be registered in the state in which the event occurred. As a result, many migrant children from other areas of India remain unregistered.
UNICEF, 2013	Supply, Incentive	2001 on	4) Costs to the health system 6) Facilitators	The initial cost of linking 212 maternity wards with 198 civil registration offices online in Pernambuco state was USD 1.5 million, for national mobilisation was BRL 5.5 million, for national campaigns was BRL 8 million, and for computers nationally was BRL 1 million (all calculated in 2008). Field testing for linking civil registration and hospital maternity wards informatically was required prior to national scale-up. Monetary incentives needed to be provided in the context of comprehensive national policy, long-term budget allocations, and joint collaboration between civil registration authorities at national and subnational levels
Upham, 2012	Supply, Demand, Incentive	1996 on	6) Facilitators	Government leadership, partnership among government agencies and academic institutions, and political advocacy facilitated the improvement and uptake of civil registration.
WHO, 2013	Supply, Demand	1997-2008	6) Facilitators	The intervention was successful because it used a combination of strategies, including the advocacy campaign, increased interpersonal communications, and the development of national partnerships. Baseline formative research, indicating that in this male-dominated culture advocacy targeted at the rights of infants instead of the rights of women would be most successful, also informed implementation

WHO, 2013	Supply, Demand	1996-2006	7) Barriers	The major barriers to advocacy included an already complex registration process, time and financial costs, lack of understanding of the importance of birth registration, and no easy way to register births occurring at the home. Various barriers for improving accessibility were limited data on the number of unregistered births to guide the programme, insufficient training of staff, lack of integral resources like stationary, and staff requiring fees for registration when legally they should be free.
WHO, 2013	Supply, Demand, Incentive	2003 on	6) Facilitators	The government's leadership role was integral to the success of these multifaceted birth and death registration interventions. Support from researchers and academic institutions conducting formative research informed the governmental programs. Finally, the success of the outreach/awareness campaigns can be partially attributed to the focus on community leaders and local grassroots organizations.
World Bank, 2015	Supply, Demand, Incentive	2007-2009	6) Facilitators	To relax legal documentation requirements, communal committees orally heard required evidence for birth and death registration and forwarded the information to the Civil Registrar for authorization and processing. Lamination machines were needed to provide birth and death certificates to remote dwellers. Additional training mechanisms, such as diploma and certificate programmes through the University of Botswana, were required for decentralisation. Campaigns required partnerships with stakeholders for technical and financial support. Multi-pronged IEC was required to reach different segments of the population (e.g. electronic media, radio, television, and speaking at public meetings convened by village chiefs and sub-chiefs was required depending on the district). Partnerships required for enforcement of requirements. For example, partnership with Ministry of Education helped facilitate enforcement of birth certificate requirement for school enrolment. Empowering UID to have uses (e.g. social protection programmes and voting) incentivised people to get birth certificates.
