# Supplementary material for: Evaluation of approaches to strengthen civil registration and vital statistics systems: A systematic review and synthesis of policies in 25 countries
Source: PLoS Med. 2019 Sep 27;16(9):e1002929. doi: 10.1371/journal.pmed.1002929 (PMC6764661; doi:10.1371/journal.pmed.1002929)
Supplement: Attachment — Submitted filename: Response to reviews.doc [file pmed.1002929.s006.doc]

Editorial team

1) Thank you for providing your PRISMA statement. Please replace the page numbers with paragraph numbers per section (e.g. "Methods, paragraph 1"), since the page numbers of the final published paper may be different from the page numbers in the current manuscript.

Authors: Thank you for this guidance. We have revised the PRISMA statement as suggested.

2) Please edit the first section of the title to be less aspirational and more descriptive of the current study.

Authors: Thank you for this suggestion. We have changed this section of the title from “Towards universal civil registration and vital statistics systems” to “Strengthening civil registration and vital statistics systems”

3) Please include the full dates of your search in the Abstract.

Authors: Thank you for this suggestion. We have included the full date of the search in the abstract in lines 42-43.

4) In the last sentence of the Abstract Methods and Findings section, please describe the main limitation(s) of the study's methodology.

Authors: Thank you for this suggestion. We have elaborated on our biggest methodological limitation, the threat of publication bias, in lines 58-60:

“The primary limitation of this systematic review was the threat of publication bias wherein many countries may not have documented their experience; this threat is most concerning for policies that had neutral or negative effects.”

5) At this stage, we ask that you include a short, non-technical Author Summary of your research to make findings accessible to a wide audience that includes both scientists and non-scientists. The Author Summary should immediately follow the Abstract in your revised manuscript. This text is subject to editorial change and should be distinct from the scientific abstract. Please see our author guidelines for more information: [https://journals.plos.org/plosmedicine/s/revising-your-manuscript#loc-author-summary](https://journals.plos.org/plosmedicine/s/revising-your-manuscript#loc-author-summary)

Authors: Thank you for this guidance. We have added this text based on the guidelines provided on pages 4-5.

6) The Abstract Conclusions, as well as the end of the discussion, should be more qualified (“Our systematic review suggests…” or similar).

Authors: Thank you for raising this oversight. We have revised the abstract as follows “Our systematic review suggests that combination policy approaches, consisting of at least a supply and demand component, were consistently associated with improved registration rates in different geographical contexts.” in lines 62-64.

7) I believe the data statement should point to the primary articles, not the SI files.

Authors: Thank you for raising this oversight. We have revised the data statement to point to the primary articles and not the SI files.

8) Line 345, please add “to our knowledge” to qualify your assertion of primacy.

Authors: Thank you for raising this suggestion. We have added this qualification to temper our conclusions.

Reviewer #1: Alex McConnachie, Statistical Review

Suther et al provides a report on published evidence of the impact of policy initiatives to improve birth and death registration; this review looks at the use of statistics in the paper.

There is not much for me to review. The figures are quite good. The lack of meta-analysis is justified. My one thought in reading the paper is whether there might be some publication bias, if those implementing unsuccessful policies are reluctant to publicise their findings, but the authors acknowledge this as a limitation of their study. Overall, I found the paper to be interesting to read, and well written.

Authors: Thank you for your careful review and kind words. We hope this article can contribute to the literature.

Reviewer #2:

Overall a well-written and interesting paper which fills a gap in current literature. A few minor points regarding specific language used below:

Authors: Thank you for your careful review and kind words.

1) Line 82-83: The sentence indicates that household surveys are mainly used to monitor registration levels. It should be highlighted that in the absence of CRVS data, household surveys are used not only for registration completeness but also for key population indicators.

Authors: Thank you for this suggestion. We have added this in lines 120-122:

“In the absence of reliable CRVS data, household surveys have become a key source of data to monitor levels and trends in births, deaths, and other core population indicators [6-9].”

2) line 85-89, it is not only about health planning but planning more broadly

Authors: Thank you for this suggestion. We have revised the text to incorporate it in line 125-128:

“Locally developed and sustainable CRVS systems can provide a legal identity from birth, the fundamental documentation to claim a nationality, and generate granular strategic information both to successfully deliver services and improve planning, budgeting, and programming for health and other sectors.”

3) line 110-110: the references about slow progress are a bit outdated (most recent 2014) there has been a lot of progress since then (particularly in South Asia). Also, is an improvement from 58-63 per cent in 15 years slow progress?

Authors: Thank you for raising these concerns. We have

(1) Added an additional reference from 2019 which summarises the status of CRVS in several low- and middle-income countries:

Suthar AB, Khalifa A, Joos O, et al. National health information systems for achieving the Sustainable Development Goals. *BMJ Open*. 2019 May 16;9(5):e027689.

(2) Added qualifers to indicate that there has been “some” progress and that the rates have increased at a “slow” rate in lines 151-152:

“Although there has been some progress in CRVS system development over the past two decades, birth and death registration rates continue to increase at a slow rate [16-18].”

4) line 245-246: registration coverage or registration completeness?

Authors: Thank you for detecting these typos. We have withdrawn “coverage” so the text simply reads “registration rates” in lines 286-287:

“Although most articles reported effects on registration rates, registration rates should be contextualised as part of CRVS business processes [78].”

Reviewer #3:

The topic of the impact of national CRVS implementation policies on outcome on birth and death registration as well as timeliness of registration is an important one as the Lancet 2007 (Who Counts) and 2015 (Counting Births and Deaths) series indicate. In the 2015 series, Phillips et al used modelling to conclude that improved CRVS performance coincided with improved health outcomes.

This MA and SR reviews the evidence on national policy interventions to improve birth and death registration. It does not attempt to correlate policy interventions with improved health outcomes.

The methodology is solid; however, the policies were heterogeneous, the sample size small, and many regions of the world were not represented in the available literature. While this is not the fault of the authors, it does limit the conclusions.

Given these limitations, I think this manuscript would better placed in a more specialized journal.

Authors: Thank you for your careful review. We hope we have addressed each of your concerns below.

Specific queries for the authors:

Making casual links between the policies and changes in birth or death rates would be challenging as policy effects must be taken into account with other country specific contexts such as political and economic instability, especially in fragile states. More context understanding would be important and strengthen this associations (as mentioned by the authors).

Authors: Thank you for raising this concern. We have revised the conclusions to ensure we are not drawing causal links from the observational studies identified in lines 62-64:

“Our systematic review suggests that combination policy approaches, consisting of at least a supply and demand component, were consistently associated with improved registration rates in different geographical contexts.”

The analysis of the operational considerations was very interesting. However, conclusions that can be drawn might be limited to the small sample size.

Authors: Thank you for raising this concern. We agree and have added this limitation in lines 311-312: “Across operational considerations, caution should be exercised in interpreting the findings due to the small sample size of articles with qualitative data.”

The applicability of one policy to another country may be limited given the differences in contexts. For example, the conclusion that a demand only policy might not be effective was based on an n=1. 1 reported this (Mongolia) where 90% coverage was already achieved.

Authors: Thank you for raising this concern. We agree and have incorporated this into the discussion in lines 317-321:

“The single article that reported on a demand-only policy did so in a context, Mongolia, where birth and death registration rates already exceeded 90% [57]. Therefore, the challenge in Mongolia was reaching the remaining few rather than increasing wide-scale access. This underscores the need to interpret the findings based on the health system, governance, and sociocultural context of contributing articles.”

Methods:

24/33 studies had no comparator group which means the effect of supply, demand, and intervention policies on birth registration was determine from small sample size (n=9). Pls address this in discussion

Authors: Thank you for this comment. We looked over the article and found that 17 articles included a comparator (see supporting information file). Three of these were excluded because they included the same time period and country as another of the inclued studies (when multiple studies included the same time period and country we chose to include the study that followed participants the longest in the figures, this is specified in lines 215-217). We agree on the need for further information and have suggested further research in this area as the final sentence of the article, lines 396-398:

“Further research using a more systematic approach to intervention design and evaluation is needed to improve the knowledge of effects, resource requirements, and acceptability of policies for strengthening CRVS.”

Similarly Figure 4 only has 5 studies reporting policy effect on death registration. What conclusions can be made from this?

Authors: Thank you for this comment. The figures summarise the quantative results from the identified studies. The interpretation of these data is in the discussion section.

How can the authors conclude a new national CRVS policy has a casual effect and how can one draw conclusions from the heterogeneous types of policy intervention and the varying country contexts? There will be so much noise from whatever else is happening in the country at the time that the actual effect of the new policy is difficult to determine.

Authors: Thank you for raising this concern. We agree and have revised the conclusions to include the word “associations” to ensure we are not drawing causal links from the observational studies identified in lines 62-64:

“Our systematic review suggests that combination policy approaches, consisting of at least a supply and demand component, were consistently associated with improved registration rates in different geographical contexts.”

This methodology does not capture migratory populations

Authors: Thank you for raising this concern. This systematic review focussed on national policies to improve birth and death registration. Countries have different policies on how migrants are registered for births and deaths and the investigators did not provide this level of detail or subanalysis in the articles. We have listed this as a gap in lines 388-389:

“Third, the articles did not provide detail on how migratory populations were registered for births and deaths and whether the findings presented were generalisable to them”

What about costing of vital records systems? The authors mention "financial"

Authors: Thank you for raising this concern. We agree that the costing of policies was a gap and have listed this in a gap of 384-385. We believe that costing entire CRVS systems, including elements outside of the policies summarised, is outside the scope of our systematic review:

“First, more information is needed on financial and human resource requirements for different policies.”

Europe not represented. Is this bc they are already have robust systems? No comments on this in paper. Even historicaly?

Authors: Thank you for raising this concern. Table 1 includes the location of included studies. Three studies were from Europe (Albania, Switzerland, and Turkey). We agree this is limited data and have listed the need for more research from this region in lines 65-66.

Is there precedent that CRVS national policies impact health outcomes (other than modelling studies)?

Authors: Thank you for this comment. This systematic review focussed on strengthening CRVS systems with respect to birth and death registration. We agree that looking at additional outcomes, such as effects of policies on health outcomes, is worthwhile and have encouraged this research in lines 389-391:

“Fourth, this systematic review focused on birth and death registration data directly collected from CRVS systems; including whether the policies had effects on additional health outcomes merits further research.”

Can Taiwan really be considered a territory of China? (Lu, 2002)?

Authors: Thank you for this query. Taiwan has not held a seat in the United Nations since China’s rights were restored in 1971 by Resolution 2758/26 (http://daccess-ods.un.org/access.nsf/Get?Open&DS=A/RES/2758(XXVI)&Lang=E&Area=RESOLUTION). Moreover, the 1979 U.S.-P.R.C. Joint Communique switched U.S. diplomatic recognition from Taipei to Beijing and recognised the Government of the People’s Republic of China as the sole legal government of China (<https://www.state.gov/u-s-relations-with-taiwan/>). We have adopted United Nations terminology for identifying Taiwan (i.e. China, Taiwan Province of China) in Table 1.

Past-present tense issues

The Village Executive Officer can access this application and view trends in notifications, and even see when a notification was made but no certificate was acquired.

Authors: Thank you for pointing out this issue. We have revised Table 1 as follows:

“The Village Executive Officer accessed this application and viewed trends in notifications, and saw when a notification was made but no certificate was acquired. “

One of the sections is termed, "Impact on birth and death registration rates

Can you make such a strong statement as to say a specific policy had a negative or positive change on birth or death registration rates? This suggests casuality. Wouldn't association be a more accurate term?

Authors: Thank you for this suggestion. We have revised the title to be “associated impact on birth and death registration rates” in line 253.
