# Supplementary material for: Evaluation of approaches to strengthen civil registration and vital statistics systems: A systematic review and synthesis of policies in 25 countries
Source: PLoS Med. 2019 Sep 27;16(9):e1002929. doi: 10.1371/journal.pmed.1002929 (PMC6764661; doi:10.1371/journal.pmed.1002929)
Supplement: Attachment — Submitted filename: Response to reviews.doc [file pmed.1002929.s007.doc]

Editorial team

- The last two points of the "What Did the Researchers Do and Find?" section of the Author Summary can be removed.

Authors: Thank you. These have been removed.

- Please add a column to table 1 to list the region each country belongs to (eg, Southeast Asia, Western Pacific, etc).

Authors: Thank you. We have added the regions.

Production team

Figures

-	Figs 1,2,3,4 appear as though they were originally made at low resolution (below 300ppi) and will appear blurry when published. This error cannot be fixed by simply increasing the resolution of your figures with image editing software. Instead, please carefully review the PLOS Figure Guidelines and provide figure files that were originally created at a minimum of 300 pixels per inch (ppi) and that are sized between 6.68cm - 19.05cm wide, and no more than 22.225cm tall (excluding any white space around your artwork).

-	The legend text in Figs 2,3,4 is below our minimum font size of 8pt and will be difficult to read when published. Please remake the text in question at a larger size or take steps to make the text readable.

- Unfortunately, if you are unable to provide a map that it created from a public domain source, we must ask that you remove it from the manuscript.

Authors: Thank you. We have redrawn the figures at 330 ppi with font sizes meeting the specifications provided. We have withdrawn figure 2 since the article’s regions are now provided in our Table 1.

Article Body

-	Please move the disclaimer from the title page to the end of the Acknowledgments section of your manuscript, with a line break between the main text of the acknowledgments and the disclaimer statement.

Authors: Thank you. We have done so.

Supporting Information

-	Please ensure that all Supporting Information files use the following format verbatim throughout the manuscript (including legends and all in-text citations): S1 Fig, S1 Table, S1 Text, S1 Data, etc. Each file type should start at 1 and increase numerically. Please note, however, that the file names themselves must use an underscore rather than a space, i.e. S1_Fig.tif, S1_Table.xlsx, S1_Text.doc, S1_Data.xlsx, S1_PRISMA_Checklist.docx, etc.

-	Please ensure that each supporting information file has a legend at the end of the manuscript file, after the Reference list.

- Your Protocol and PRISMA checklist will need to be published with your article. Please therefore ensure that they have the file type 'Supporting Information' rather than file type ‘Other’ in the system’s file inventory. Please ensure they meet naming requirements.

Authors: Thank you. We have done so.
